# Giant Colonic Lipoma Remains a Surgeon's Domain: A Report of Two Cases

**DOI:** 10.7759/cureus.43488

**Published:** 2023-08-14

**Authors:** Jaai Vishnu, Nagaraj Kapil, Vaishnavi Pe, Pooja Patnaik

**Affiliations:** 1 General Surgery, Meenakshi Medical College Hospital and Research Institute, Kanchipuram, IND; 2 Surgical Gastroenterology, Meenakshi Medical College Hospital and Research Institute, Kanchipuram, IND

**Keywords:** fat density lesion, endoscopic mucosal dissection, intususseption, right hemicolectomy, colonic lipoma

## Abstract

Colonic lipomas are rare benign submucosal tumors that are mostly asymptomatic. With increasing size, they may develop symptoms and complications. The acute presentation may be intestinal obstruction secondary to intussusception or gastrointestinal bleeding. The chronic presentation may be subtle and mimic a colonic malignancy. Symptoms include altered bowel habits, abdominal pain, lower gastrointestinal bleeding, and weight loss. Diagnostic evaluation includes advanced imaging such as Computed Tomography, Magnetic Resonance Imaging, and Endoscopy. With the advent of endoscopic submucosal dissection techniques, the therapeutic capabilities of endoscopy have expanded over the decade. However, surgical interventions were reserved for large, symptomatic lipomas, and resection varies from segmental colonic resection to hemicolectomy. Size and clinical presentation determine the therapeutic approach. We, with this, report two cases of giant colonic lipoma in the right colon causing a colo-colic intussusception.

## Introduction

Lipomas in the gastrointestinal tract are uncommon and can occur anywhere along its length. In the large intestine, their incidence ranges from 0.2% to 4.4%. Gould et al. stated that they usually occur in the fifth decade of life and affect women more than men [1]. The commonest site of colonic lipomas is considered to be the ascending colon (45%), followed by the sigmoid colon (30.3%), descending colon (15.2%), and transverse colon (9.1%). Most are asymptomatic. The lipomas of the gastrointestinal tract that measure greater than 2 cm may present with abdominal pain, diarrhea, or constipation. The ones greater than 4 cm in size lead to obstruction and intussusception. Computed tomography (CT) scan and colonoscopy are reliable diagnostic tools. The lipomas greater than 2 cm can be resected endoscopically with good remission rates [2]. Surgical management is preferred when they present with complications such as intussusception, obstruction, or bleeding. We present two cases of subacute intestinal obstruction due to intussusception caused by giant colonic lipoma in the right colon and its management.

## Case presentation

Case 1

**Figure 1:**
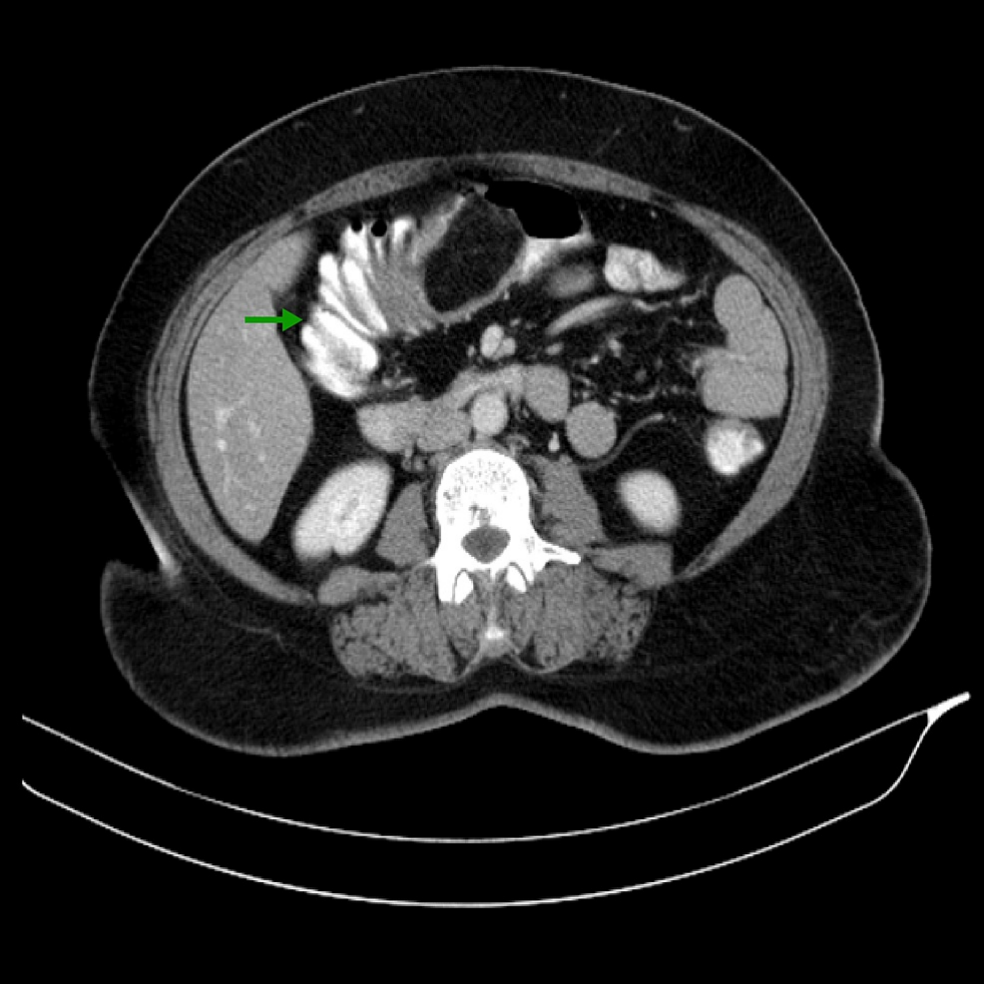
CT abdomen (axial section) The image shows fat density polypoidal lesion arising from ascending colon, prolapsing into the transverse colon (green arrow).

A 45-year-old female presented with complaints of right-side lower abdominal pain, abdominal distention, and intermittent episodes of vomiting for 15 days. She was able to tolerate liquids only. She underwent imaging, given her alarming symptoms. Her CT scan (Figure 1) revealed a fat-density polypoidal lesion measuring approximately 8x3 cm arising from the cecum and ascending colon prolapsing into the transverse colon with haustral crowding and thickening suggestive of intussusception. Small sub-centric regional lymph node enlargement was also found. Colonoscopy was suggestive of intussusception of a polypoidal submucosal lesion with normal overlying mucosa.

**Figure 2:**
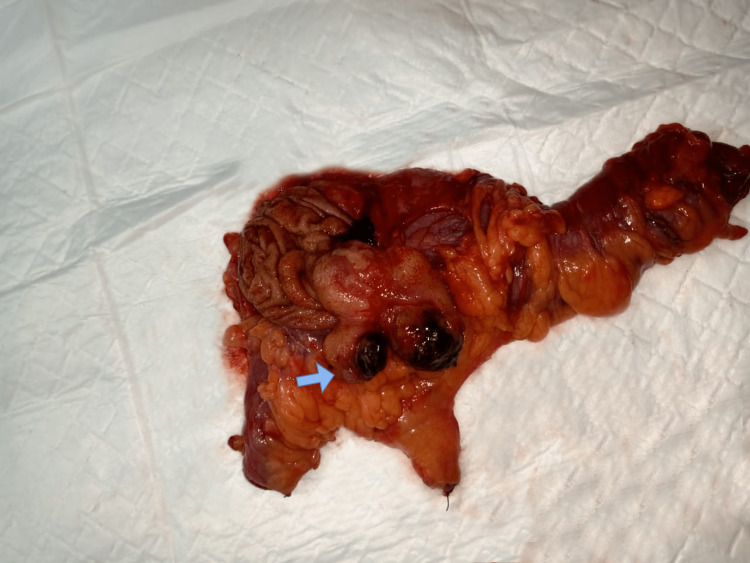
Right hemicolectomy product Three polypoid submucosal lesions arising from the ascending colon, with mucosal necrosis (blue arrow).

She was planned for an open right hemicolectomy. The examination of the specimen showed three polypoidal lesions arising from the ascending colon. Incising the mucosa revealed three submucosal lipomas (Figure 2). The histopathological study report revealed well-circumscribed adipose tissue in the submucosa, overlying mucosal necrosis, atypical stromal cells, and florid vascular proliferation. Carcinoembryonic antigen (CEA) was 1.5 ng/ml. The patient was followed up for three months. She had no complications on follow-up.

Case 2

**Figure 3:**
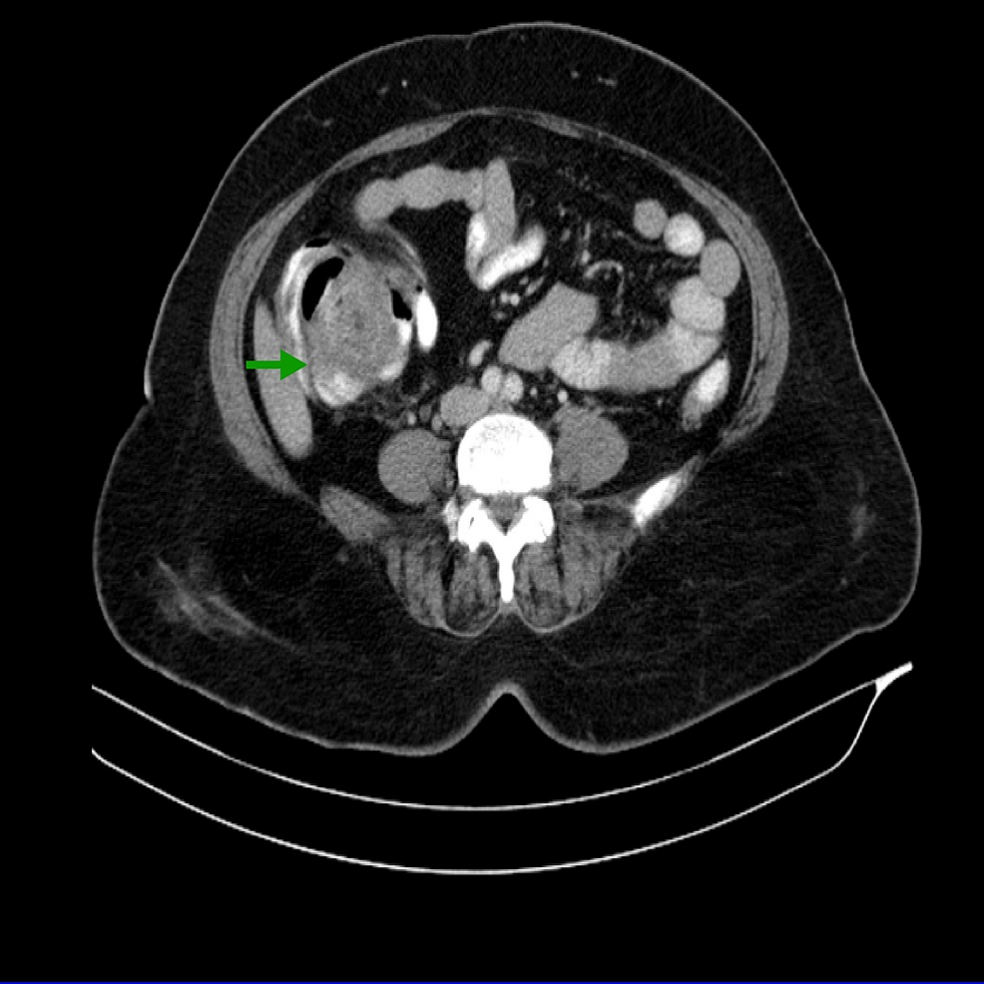
Abdominal CT scan image (axial section) The image shows a heterogenous soft tissue lesion causing intussusception, involving the cecum (green arrow).

The second case was a 53-year-old male who presented with diffuse abdominal pain and vomiting for one week. He also had abdominal distension and constipation. He underwent imaging (Figure 3) and colonoscopy, suggesting intussuscepting giant colonic lipoma. The proximal colon could not be visualized during the colonoscopy due to the intussusception. An open right hemicolectomy was done. The specimen was cut opened and examined; the ascending colon had one massive polypoidal mass measuring 5x3 cm in dimension. After incising the mucosa, submucosal lipoma was seen with homogenous fat with a fibrous capsule surrounding it. The histopathology study revealed well-circumscribed adipose tissue in the submucosa with fibrosis, overlying mucosal ulceration with florid vascular proliferation. CEA level was 1.1 ng/ml. The patient was followed up for three months. He did not present with any complications on follow-up.

## Discussion

Colonic lipomas are sporadic benign tumors of the large bowel. It is considered the second most common benign tumor of the colon, with an incidence between 0.2 and 4% [3]. 90% of the lipomas originate in the submucosal layer. Colonic lipomas present as a sessile polypoidal mass in 90 percent of cases. Lipomas larger than 4 cm are considered giant colonic lipomas and cause symptoms in 75 percent of cases. They are more commonly found in women with a peak incidence in their fifth decade [4]. The most common symptoms are abdominal pain, lower gastrointestinal bleeding, alteration in bowel habits, weight loss, obstipation, intermittent vomiting, etc. Lipomas of the colon may have ulcerated or necrotic overlying mucosa due to chronic pressure effects caused by intussusception, traction, and violent peristalsis, especially if they are pedunculated lipomas. Adult colonic intussusceptions are also caused by primary carcinoma in 65 to 75 percent of the cases [3]. The presence of necrotic mucosa, ulceration, and bleeding makes the differentiation from a malignant lesion at colonoscopy difficult. The risk of colonic lipoma developing into a carcinoma is very low but not unheard of [5]. Normal overlying mucosa usually points to a benign etiology.

Imaging modalities and colonoscopy are the mainstays of diagnosis of colonic lipomas. A lipoma has a uniform appearance on a CT with fat-equivalent density and a smooth border (−40 to −120 Hounsfield units). The sensitivity of a CT scan to diagnose intussusception is 71.4%-87.5%, and its specificity is 100% in adults. Intussuscepted lipomas may not demonstrate normal fat attenuation and may have a heterogeneous appearance reflecting the degree of infarction and fat necrosis, leading to a diagnostic dilemma. Magnetic resonance imaging is an alternative in the diagnosis, which shows a signal intensity characteristic of adipose tissue on T1 weighted and fat suppression images [6]. Barium enema shows a filling defect but is less used for diagnosis given the advent of advanced imaging [6].

Colonoscopy is mandatory for diagnosis and therapeutic for colonic lipomas less than 2cm. Lipomas appear as a well-delineated, soft, sessile, or pedunculated mass in a colonoscopy. Sometimes ulceration and erythema can be seen on the mucosa, giving the impression of a malignancy. The three critical endoscopic signs of colonic lipoma are the 'cushion sign' (probing the polyp with a closed biopsy forceps will often yield a pillow-like indentation), 'tenting effect' (grasping the overlying mucosa with biopsy forceps presents a tent-like appearance), and the 'naked fat sign' (biopsies may result in extrusion of yellowish fat). Endoscopic ultrasound helps differentiate a lipoma from other submucosal lesions like schwannoma and leiomyoma. Extension of the lesion into the deeper of the submucosa can also be detected by endoscopic ultrasound [7].

Endoscopic resections like Endoscopic Mucosal Resection (EMR) and Endoscopic Submucosal Resection (ESD) have expanded the size criterion. Endoscopic deroofing has also been reported, allowing spontaneous lipoma expulsion just by deroofing the mucosa over the summit [8]. Application of endoscopic procedure is limited to tumors arising from submucosa, and risks of perforation increase exponentially with the deeper layers involved. Experience with colonic ESD has been scarce worldwide. Moreover, colonoscopic resections are avoided in acute presentation due to the inability to prepare the bowel and questionable viability of the involved segment in intussusception. The site also plays a significant factor as right-sided colon lesions are more prone to perforation than left-sided colon lesions.

Colon lipomas >2 cm are considered for surgery due to the high risk of complications, such as bleeding and perforation with endoscopic resection. Surgery is the mainstay of treatment for lipomas causing complications, those with increased risk for endoscopic resections and suspicion of malignancy. Standard colonic resections are preferred over segmental colonic resections. Standard hemicolectomy is more standardized and practiced more frequently. Segmental colonic resections have fallen out of favor because the colonic blood supply is variable. Owing to the sparse blood supply in the watershed areas of the colon, segmental colectomy has a high chance of anastomotic leaks due to postoperative ischemia. It is not recommended to deviate from the standard procedure, i.e., hemicolectomy (open or laparoscopically). We suggest a D2 lymphadenectomy if there are doubts about diagnosis in emergencies where colonoscopy was not feasible. One such scenario is an intussuscepting giant lipoma with heterogeneous imaging features wherein a colonoscopy was deferred because of acute presentation.

Postoperative complications, like intra-abdominal abscesses, postoperative ileus, wound infection, and respiratory failure occur more often following open procedures. Postoperative chronic pain is less likely to occur in patients undergoing laparoscopic resection than those undergoing open laparotomy [9]. The surgeon's experience and feasibility towards the procedure play a pivotal role in choosing the approach of surgery.

## Conclusions

Colonic lipomas, greater than 2 cm in size, may present with symptoms. CT, MRI, and colonoscopy aid in the diagnosis of colonic lipomas. Endoscopic resection is the preferred method for treating both incidental and symptomatic lipomas, but there are limitations. Surgery remains the choice of treatment for lipomas having acute presentations. Based on imaging findings, an appropriate lymphadenectomy may be included when there is suspicion of malignancy in acute presentation.
